# Genome Size Variation across a Cypriot Fabeae Tribe Germplasm Collection

**DOI:** 10.3390/plants12071469

**Published:** 2023-03-27

**Authors:** Iliana Charalambous, Nektaria Ioannou, Angelos C. Kyratzis, Dimitrios Kourtellarides, Marianna Hagidimitriou, Nikolaos Nikoloudakis

**Affiliations:** 1 Department of Agricultural Science, Biotechnology and Food Science, Cyprus University of Technology, 3036 Limassol, Cyprus; im.xaralampous@edu.cut.ac.cy (I.C.); nk.ioannou@edu.cut.ac.cy (N.I.); 2 Vegetable Crop Sector, Agricultural Research Institute-Ministry of Agriculture, Rural Development and Environment, 1516 Nicosia, Cyprus; akyratzis@ari.moa.gov.cy (A.C.K.); dkourtellarides@ari.moa.gov.cy (D.K.); 3 Department of Biotechnology, Agricultural University of Athens, 10561 Athens, Greece; marianna@aua.gr

**Keywords:** C-value, DNA content, genetic resources, flow cytometry, legumes

## Abstract

DNA content is an important trait linked to the evolutionary routes of taxa and often connected to speciation. In the present study, we studied C-values variation across the Cypriot Fabeae gene pool. Several hundred plants (*Vicia* spp., *Lens* spp., *Pisum* spp.) were sampled across Cyprus. Accurate estimates were established by flow cytometry and propidium iodine staining for 155 discrete populations/accessions. A ten-fold variation was detected across lineages with 1C DNA content varying from 1.584 pg for *V. cretica* (ARI02420) to 13.983 pg for *V. faba* (ARI00187). In general, flow cytometry was precise for the characterization of species, even though there were instances of genome overlapping across taxa. Most analyses in the current work refer to species that have not been characterized before by flow cytometry (or any other DNA content estimation method). Still, a correlation to C-values previously reported in Kew Plant DNA C-values database was attempted. A high degree of correlation except for *V. dalmatica* was established. The evaluation of genome size trait in relation with the Fabeae phylogeny, revealed that *Pisum* and *Lens* genera were rather homogenous, but an astonishing fluctuation was shown for *Vicia* spp. Moreover, it was established that genome up- or down-scaling was not directly linked to speciation drivers. The genomic size measurements presented here could deliver extra quality control for the identification and characterization of taxa in germplasm collections, particularly in cases where species share morphological characters.

## 1. Introduction

The inconsistency of DNA concentration within a haploid nucleus (C-value) and the functional complexity of eukaryotic organisms has been recognized as the C-value paradox [1]. The fact that the DNA amount is not correlated to the complexity of an organism or the number of required genes, despite initial theories, has been an enigma, perplexing geneticists across the decades. Nowadays, complementary to last eras’ C_0_t analysis (a technique based on reassociation kinetics of DNA fragments), next generation sequencing (NGS) has provided solid evidence that genome augmentation is due to repetitive DNA sequences, formerly believed to be ‘junk DNA’ [2].

Angiosperms (flowering plants) are generally accepted as ideal candidates to study the influence of such repetitive sequences on the evolution of genomes [3] since there is an astonishing difference across genome sizes, spanning over three orders of magnitude (starting from approximately 0.59 Gbp/1C) in the carnivorous plant *Genlisea tuberosa* Rivadavia, Gonella & A. Fleischm [4] to 148.90 Gbp/1C in *Paris japonica* Franchet [5]. Even though C-values greatly fluctuate across taxonomic families, there is general uniformity within families, tribes, or species.

Nonetheless, this is not the case for the legume family (Leguminosae) where great dissimilarities of genome size are reported; C-values fluctuate from approximately 400 Mbp to more than 26,000 Mbp (per 1C) [6]. In particular, smaller genomes (0.34 pg/1C) have been reported for *Trifolium ligusticum* Balb. ex Loisel. [7], while *Vicia faba* L. has been recognized as having the largest genome (27.40 pg/1C) [8]. Ancestral forms of legumes (such as *Trifolium* spp) are alleged to have had smaller genomes, while DNA content across the Fabeae tribe has staggeringly increased after the divergence from common progenitors [6]. The Fabeae tribe contains economically/agronomically significant taxa for human, as well as animal, consumption (such as *Lathyrus* L., *Lens* Mill., *Pisum* L. and *Vicia* L.). The accumulation of available molecular data for Fabeae, such as genetic linkage maps [9,10,11,12], genome sequencing [13,14,15], and diversity arrays technology (DArT) [16] has provided valuable insights for these genetic resources. Still, a basic understanding regarding the nature of a given genome necessitates information concerning the DNA amount and must be considered as a critical feature in any inclusive program for comparative genomic investigations [17]. Nonetheless, despite the fact that hundreds of different taxa have been reported in the literature for Fabeae taxa, very few data regarding genomic size are reported and available [18].

The first evaluation on genome size for Fabeae was reported by Rees et al. [19] for *V. sativa* (1.13 pg). The genus *Pisum* comprises three species: *P. abyssinicum* (Abyssinian pea), *P. fulvum* (red yellow pea), and *P. sativum* (garden pea), with two subspecies, wild *P. sativum* subsp. *biflorum* and cultivated *P. sativum* subsp. *sativum* [20,21]. The genus *Lens* includes seven taxa clustered in four species namely: *L. ervoides*, *L. lamottei*, *L. nigricans*, *L. culinaris* (*L. culinaris* subsp. *culinaris*), *L. odemensis* (*L. culinaris* subsp. *odemensis*), *L. orientalis* (*L. culinaris* subsp. *orientalis*), and *L. tomotosus* (*L. culinaris* subsp. *tomotosus*) [22]. Oppositely, the *Vicia* genus is a multitudinous taxonomical unit [23], having extensive germplasm resources spanning to 216 species [24]. Until present-day, 84 *Vicia*, 3 *Pisum*, and 2 *Lens* species have had their nuclear DNA content assessed (https://cvalues.science.kew.org/ (accessed on 3 March 2023)), which signifies a limited amount considering the large number of designated Fabeae taxa.

The study of the genome is a significant approach to comprehend the evolutionary mechanisms of plant species [25]. Yet, the knowledge of Fabeae diversity has mainly focused on chromosomal analyses and/or morphological characters [26,27,28,29,30,31,32,33,34,35,36,37]. Moreover, most reports on Fabeae C-value have been provided by Feulgen microdensitometry. In particular, 83 reports for *Vicia* spp. prime estimates (out of 86) resulted from Feulgen densitometric analysis (https://cvalues.science.kew.org/ (accessed on 3 March 2023)). Indeed, historically in the Kew Plant DNA C-values database, DNA content was regularly assessed by means of Feulgen microdensitometry [38]. Nevertheless, this method is quite slow, laborious, and frequently has limited precision [39]. Due to its increased applicability, accuracy, high throughput, and speed, flow cytometry has gained attention for genome size estimation. [40]. As a result, nowadays flow cytometry has become the ‘gold standard’ methodology employed for calculating C-values and GC content across lineages [41,42]. Accordingly, out of the 12,273 prime estimates registered in the Kew Plant DNA C-values database, 8643 have been calculated via flow cytometry (database accessed in February 2023).

Cyprus is an island located between three continents, a hotspot of biodiversity and within the center of Fabeae taxa origin/domestication [43]. Geographical isolation in correlation to different microclimates, as well as the presence of civilization, trade, and agriculture since antiquity, has contributed to the formation of several unique genotypes across different local crops [44,45,46,47,48,49]. Hence, under an evolutionary prism, and in terms of genetic resources exploration/exploitation, there is a scientific interest for characterizing these indegenous genotypes and the Cypriot gene pool.

The aims of the current study were to provide information in the complex taxonomic Fabeae tribe by delivering precise estimations of the genome size of 29 species (155 accessions/465 plants) collected across Cyprus, the Southernmost European boarder. Multiple accessions per species were employed in order to unravel possible evolutionary insights of genome size, and to assess inter- and intra-genome size variation. Additionally, flow cytometry was evaluated as an analytical method for use in the Cypriot legume germplasm classification.

## 2. Materials and Methods

### 2.1. Plant Material

**Figure 1:**
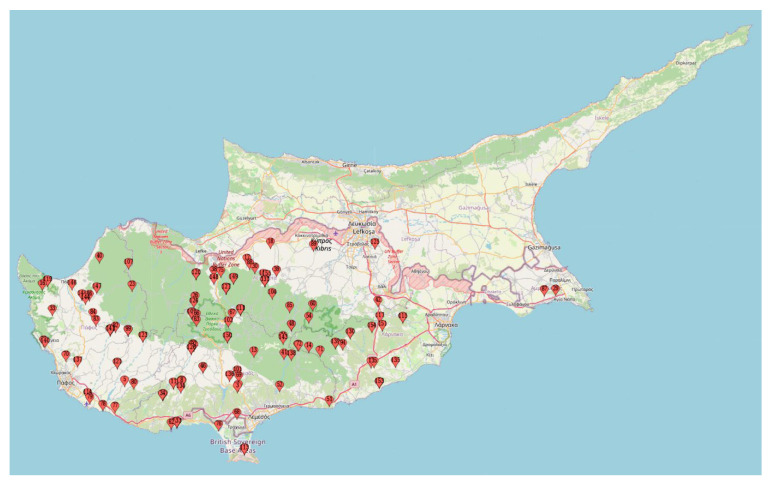
Distribution of sampling sites across Cypriot regions. In total, 155 Fabeae populations (belonging to 29 species) were collected.

For the collection of the plant material, a two-year expedition (2019–2020) was directed across regions of Cyprus (Figure 1). Plants were marked in situ (during the flowering period), identified following the flora of Cyprus keys [50,51], and pods from multiple plants across 155 Fabeae populations (*Vicia* spp., *Lens* spp. and *Pisum* spp.) were collected (Supplementary Table S1). Seeds were physically purified and deposited to the Cypriot Genebank/Herbarium (Agricultural Research Institute). Subsequently, seeds were dried to a humidity percentage of approximately 10% and stored at −20 °C. Taxa used as internal flow cytometry standards [52] were provided by Prof Doležel and the Centre of Plant Structural and Functional Genomics (Šlechtitelů 31, Holice, 779 00 Olomouc, Czechia). All internal standards used [*Solanum lycopersicon* cv. Stupické polní tyčkové rané (1C = 0.98 pg), *Glycine max* cv. Polanka (1C = 1.25 pg), *Zea mays* cv. CE-777 (1C = 2.715 pg), *Pisum sativum* cv. Ctirad (1C = 4.545 pg), and *Secale cereale* cv. Dankovské (1C = 8.095 pg)] were designated based on genomic size proximity to Fabeae samples but with no-overlapping 2C and/or 4C-values.

### 2.2. Sample Preparation

Ten seeds per accession were treated by mechanical scarification and germinated in petri dishes containing moist Whatman^®^ cellulose filter papers. Plantlets were transferred to pots containing a commercial peat/perlite mix and grown in walk-in cabinets at 20–25 °C, under a 16 h day/8 h night photoperiod. Fresh and healthy leaf tissues were collected from young plants (three discrete plants for each accession were used for sampling) and kept between humid paper towels in a fridge until chopping (within the day). A leaf area (roughly 0.5 cm^2^) of samples and standards were chopped together using a sterile double-edge razor blade for 5 to 10 s in a petri dish (placed on top of ice). Tissues were submerged at all times in one mL of pre-chilled Lysis buffer-LB01 [15 mM Tris, 2 mM Na_2_EDTA, 0.5 mM spermine tetrahydrochloride, 80 mM KCl, 20 mM NaCl, 0.1% (*v*/*v*) Tween-20, 50 µg/mL propidium iodide (PI), 50 µg/mL RNase, and 0.1% ß-mercaptoethanol] as previously reported [53]. Homogenates were passed through 50 μm Celltrics nylon filters (Sysmex, Lincolnshire, IL, USA) into 1.5 mL Eppendorfs and kept at 8 °C for 20 min to enhance staining. In total, 114 *Vicia* spp., 28 *Lens* spp. and 13 *Pisum* spp. accessions were analyzed (Table 1).

### 2.3. Flow Cytometry

C-values were assessed utilizing an Accuri C6 flow cytometer (Accuri Cytometers, Inc., Ann Arbor, MI, USA), following a previously described procedure [55]. Analysis was based on light-scatter and fluorescence signals emitted from a 20-mW laser illumination at 488 nm. The precision of the cytometer was established using 6-peak Spherotech fluorescent beads, as indicated by the vender (CFlow User Guide, Accuri, Franklin Lakes, NJ, USA). Double threshold levels were determined (80,000 on FSC-H and 1000 FL-2) to exclude irrelevant debris from detection. Fluidics were set on slow, and measurements were accumulated to an overall number of 3000 nuclei. The areas of nuclei were diagonally gated using an FL3-A/FL2-A plot and peaks were displayed via a count events/FL2-A plot. For each accession, three different measures were performed for three consecutive days. Replicates were well reproducible having minor systematic errors. Flow files were exported and analyzed using Modfit LT version 5.0 (Verity Software House, Topsham, ME, USA) to identify modelled peaks and calculate C-values. The flow histograms displayed crisp peaks, having low coefficient of variation (<5%).

### 2.4. C-Value Character Evolution

Fabeae sequences for internal transcribed spacers (ITS1-5.8S-ITS2) were identified using the nucleotide database of NCBI (https://www.ncbi.nlm.nih.gov/nucleotide/ (accessed on 3 March 2023)). Twenty-seven discrete sequences were selected and downloaded in Fasta format. Following acquisition, a multiple alignment was conducted (using the MUSCLE algorithm/Gblocks alignment curation). For the construction of the phylogenetic tree, a maximum likelihood approach was selected and implemented using a PhyML approach. The single dendrogram produced was visualized with TreeDyn and exported as a nexus file. The mesquite V3.70 suite (www.mesquiteproject.org (accessed on 3 March 2023)) was employed to embed C-values as a continuous character, and to perform parsimony analysis for linking DNA content to the Fabeae evolutionary history (using the nexus dendrogram file as an input).

### 2.5. Statistical Analyses

C-value means and standard deviations were calculated for each species (across accessions) and reported in Table 1. To perform a linear regression, residuals were used to examine the uniformity of variance and relevant fit to a normal distribution (Supplementary Figure S1). Differences across species, considering accessions as replicates, were analyzed using a one-way ANOVA test and two post-hoc analyses: Tukey’s honest significant difference (HSD) test, and least significant difference (LSD) test. Rstudio (Version 1.1.463) and the agricolae package were employed for the analyses. Three biological replications (nine discrete plants within the accession) were employed for the analysis for species for which only one population was found in Cyprus.

## 3. Results

**Figure 2:**
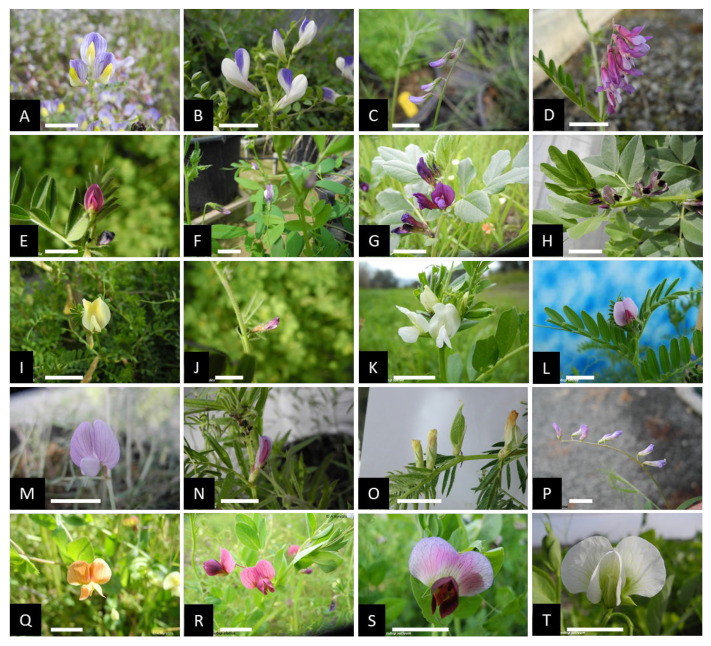
Illustration of floral diversity in the Fabeae tribe. (**A**) *V. lunata*; (**B**) *V. cypria*; (**C**) *V. laxiflora*; (**D**) *V. Villosa*; (**E**) *V. angustifolia*; (**F**) *V. bithynica*; (**G**) *V. narbonensis*; (**H**) *V. faba*; (**I**) *V. hybrida*; (**J**) *V. lathyroides*; (**K**) *V. sativa* subsp. *sativa*; (**L**) *V. sativa* subsp. *sativa*; (**M**) *V. peregrina*; (**N**) *V. amphicarpa*; (**O**) *V. lutea*; (**P**) *V. palaestina*; (**Q**) *P. fulvum*; (**R**) *P. sativum* subsp. *biflorum*; (**S**) *P. sativum* subsp. *sativum*; (**T**) *P. sativum* subsp. *sativum*. scale bar: 1 cm (all pictures © Angelos kyratzis).

**Figure 3:**
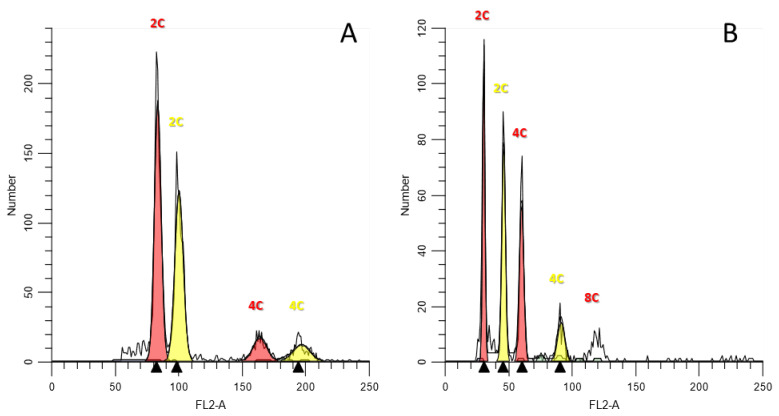
(**A**) FL2-A fluorescence histograms of *Zea mays* cv. CE-777 (red peaks) and *Pisum sativum* subsp. *sativum* ARI00308 (yellow peaks) with diploid (2C) and tetraploid (4C) nuclei. (**B**) FL2-A fluorescence histograms of *Zea mays* cv. CE-777 (yellow peaks) and *Vicia lunata* ARI01343 (red peaks) PI-stained nuclei. Besides diploid (2C) and tetraploid (4C) cells, nuclei with a higher ploidy level (8C) were also detected.

For the present study, several hundred Fabeae plants were collected across Cyprus (Figure 1). For species identification and characterization, keys reported in Meikle [50] and ‘the flora of Cyprus’ site (https://www.flora-of-cyprus.eu/ (accessed on 3 March 2023)) were followed. The Cypriot Fabeae germplasm was found rich in diversity (Figure 2) and morphological characters allowed the unambiguous identification of 29 species. Three plants from each population/accession (totaling 455 plants) were co-analyzed by means of flow cytometry and PI staining. Estimated C-values across taxa were calibrated to the genome size of proper reference internal standards (Table 1). Across all cases, the 2C peak (FL2-A axis) of each standard was detected within the 2C and 4C peak range of the Fabeae sample, or vice versa, contributing to precise DNA content estimations (Figure 3).

The FL2-A histograms of nuclear DNA content generally displayed a single distinct peak, matching to the G1 phase nuclei (2C-value), and a lower peak corresponding to G2 phase nuclei (4C-value). The analysis of distributions revealed that the vast majority of nuclei were at the G0/G1 cell cycle (Figure 3A). In some instances, higher than 4C ploidy levels were recorded and assigned to endopolyploidy cells (Figure 3B). High-resolution histograms were generated across replicates having a 2C peak coefficient of variation (CV) repeatably lower than 5%. Average CVs between biological replications on three consecutive days (using the same buffer lot) was also low (approximately 1.5%), suggestive of accurate measurements.

The 1C genome size across 155 Fabeae accessions was estimated (on average) for three consecutive days (Supplementary Table S1). It was determined that mean 1C genomic size for individual taxa varied almost ten-fold, ranging from 1.584 ± 0.007 pg for *V. cretica* (ARI02420) to 13.983 ± 0.046 pg for *V. faba* (ARI00187). The lowest CV across accessions was detected for *V. dalmatica* ARI01835 (0.08), and the higher for *V. sativa* subsp. *sativa* ARI00307 (3.82).

DNA 1C-values from discrete accessions (spanning from 1 to 17) were subsequently used in order to calculate mean genomic size and standard deviation at the species level (Table 1). Among species, the most homogenous genome size was recorded for *V. laxiflora* having a CV of 0.106, while the greatest heterogenicity was found in *V. villosa* subsp. *eriocarpa* that had a CV of 4.114.

**Figure 4:**
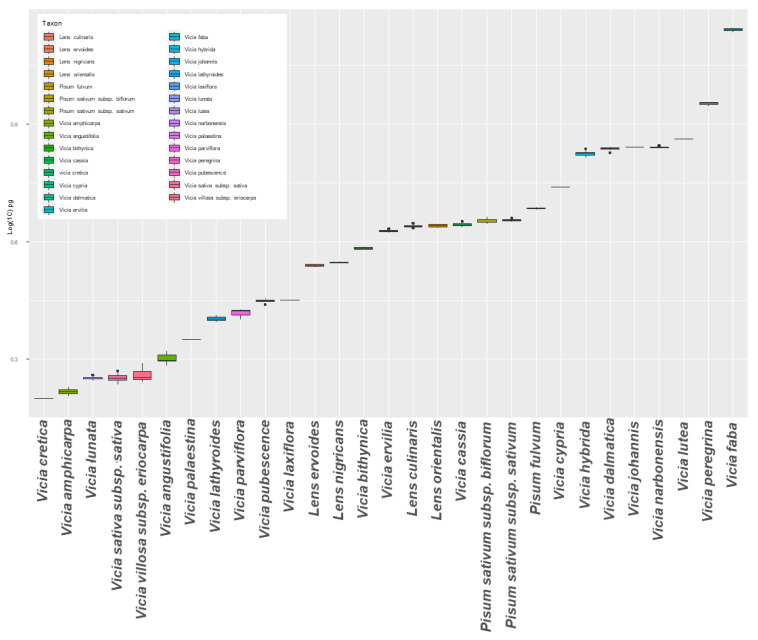
Box plots of mean 1C-values (logarithmic scale) within species. Horizontal lines depict the median value. Boxes below and above the mean line designate quartiles. Lines at the whiskers’ margins (vertically) identify extreme data points, and circles indicate the outliers.

The residuals of a model fitting were reviewed to test for equal variances across accessions (Supplementary Figure S1). It was established that systematic bias regarding variances was not evident; consequently, equal variances were assumed for post hoc analyses (HSD and LSD tests). The box plot of increasing genome sizes (Figure 4) depicted that within species DNA content variation was frequently insignificant; hence, taxa were correctly assigned to species. *Pisum fulvum* was clearly distinguished from *P. sativum* subsp. *biflorum* and *P. sativum* subsp. *sativum*. Still, across several species, post hoc analyses revealed that there is genomic size overlapping; thus, C-values cannot always be used as stand-alone traits for species assignment. Particularly, *V. dalmatica*, *V. johannis*, and *V. narbonensis* were assigned at the same genomic content group (Table 1; Figure 4). *Lens ervoides* and *L. nigricans* were also of equal genomic size, but these were clearly demarcated from *L. culinaris* that had a larger value. Also, *V. laxiflora* and *V. pubescence* were found comparable, having an average C-value of approximately 2.8 pg/1C. *Vicia lunata*, *V. sativa* subsp. *Sativa*, and *V. villosa* subsp. *eriocarpa* had similar genomic sizes (Table 1).

**Figure 5:**
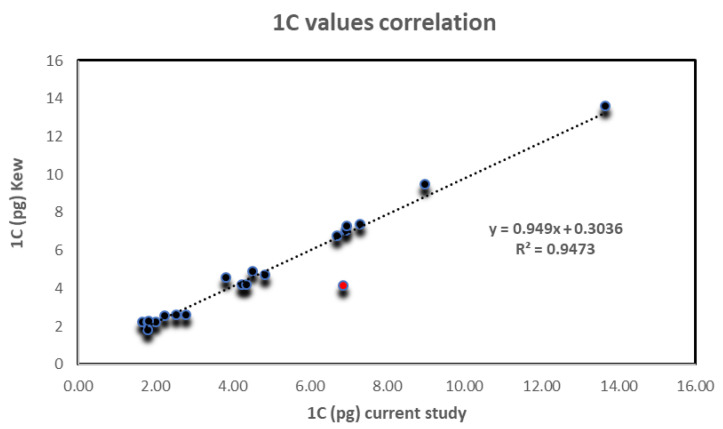
C-values correlation across the 20 common taxa analyzed in the current study that are also presented in the Kew Plant C-values database (https://cvalues.science.kew.org/ (accessed on 3 March 2023)). Data were highly correlated (R^2^ ≈ 0.95) across species. A notable departure was found merely for *Vicia dalmatica* (depicted as a red circle).

The current study reports novel genomic estimations of Fabeae taxa using flow cytometry. Still, a correlation to previously reported C-values was attempted (Figure 5). It was established that 1C-values presented here agree with previously published values (obtained with flow cytometry as well as Feulgen cytophotometry). Nevertheless, we found that the Feulgen technique tends to be equivalent when compared to PI flow cytometry (Table 1). Despite small discrepancies, a highly significant correlation was established across the 20 common species analyzed. Remarkably, the only substantial variance was observed for *V. dalmatica* where we recorded an almost 50% greater value than previous reports. Interestingly, *V. dalmatica* is a perennial plant in Cyprus and can only be found at high altitude habitats (above 800 m).

**Figure 6:**
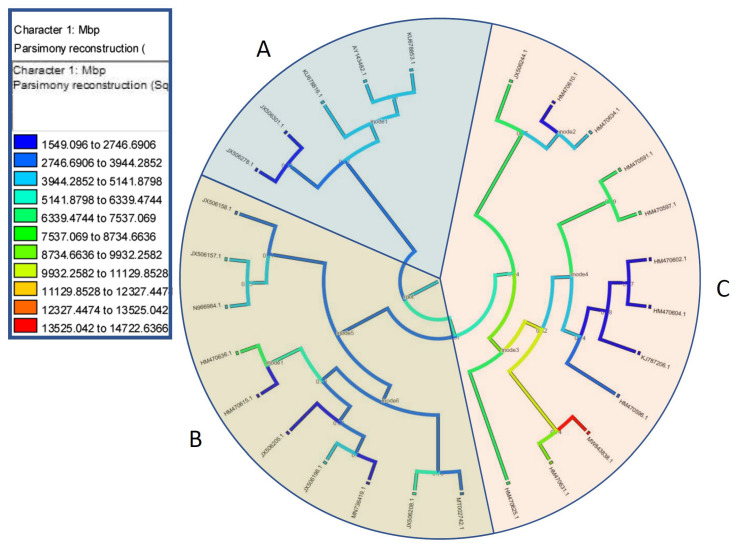
Discrete parsimony optimization of C-values (Mbp) in the Cypriot Fabeae tribe analyzed with the mesquite software. Reconstruction of ancestral states for C-values on an ITS1-5.8S-ITS2-based phylogenetic tree. Blue and cyan branches symbolize moderately small genome DNA content while green, orange, and red designate, respectively, medium-sized and larger genomes. NCBI nucleotide accessions correspond to the following taxa: JX506157.1; *L. culinaris*, MT002742.1; *L. ervoides*, JX506158.1; *L. nigricans*, JN966984.1; *L. orientalis*, KU678816.1; *P. fulvum*, KU678853.1; *P. sativum* subsp. *biflorum*, AY143482.1; *P. sativum* subsp. *sativum*, HM470604.1; *V. amphicarpa*, KJ787206.1; *V. angustifolia*, HM470596.1; *V. bithynica*, JX506196.1; *V. cassia*, JX506205.1; *V. cretica*, JX506208.1; *V. cypria*, HM470636.1; *V. dalmatica*, HM470634.1; *V. ervilia*, MW843838.1; *V. faba*, HM470625.1; *V. hybrida*, HM470597.1; *V. johannis*, HM470610.1; *V. lathyroides*, JX506301.1; *V. parviflora*, JX506244.1; *V. lutea*, HM470591.1; *V. narbonensis*, MN736419.1; *V. palaestina*, HM470631.1; *V. peregrina*, JX506278.1; *V. pubescens*, HM470602.1; *V. sativa* subsp. *sativa*, HM470615.1; *V. villosa* subsp. *eriocarpa*.

A discrete parsimony optimization analysis was subsequently performed using the mesquite software suite. The ancestral character evolution projection indicated that C-values of Fabeae genome size followed an increasing rather than decreasing pattern. Moreover, substantial genomic augmentation mainly occurred in *V. faba* and *V. peregrina* as well as in taxa grouped to the C-clade (Figure 6). Nonetheless, it seems that the Fabeae tribe also had several independent ‘upsizing’ events across other lineages (A and B). Nonetheless, genome size was not found to be associated with any phylogenetic lineage or evolutionary driver.

## 4. Discussion

Legumes include crops that are cultivated on a significant area of global arable land (12–15%) and account for approximately 27% of the world’s primary crop production [56]. Within the leguminosae family, the Fabeae tribe is constituted by five genera, comprising 380 species which hold agricultural significance. These mainly refer to lentil (*Lens culinaris*), broad bean (*Vicia faba*), domesticated vetches (mainly *Vicia sativa* and *Vicia ervilia*), and pea (*Pisum sativum*) [57]. Besides the current significant agronomic status, these species were also of immense importance since antiquity and have been utilized alongside major domesticated crops (barley, wheat and flax) in the fertile crescent [58]. Faba beans specifically have been used, not only as food, but were intertwined with the archaic Mediterranean history and tradition [59]. In the birthplace of democracy (Ancient Greece), broad beans were used for casting votes (white beans signified a positive vote, and black/brown beans stood for a negative vote). Even today, the expression ‘koukiá’ (meaning ‘broad beans’ in Greek) is informally used, denoting votes. Faba beans were also used as ‘food for the dead’ in the Lemuria festival throughout the classical Roman era.

Nowadays, Fabeae taxa span virtually to a worldwide grid, but the main source of genetic diversity is still found within Eastern Mediterranean that is considered the center of origin/domestication for these lineages [24]. Cyprus is within the domestication region and a geographical boundary/cut-off point for three continents (Europe, Africa and Asia). Its position and diverse microclimatic types has established the island as a hot spot for plant biodiversity, despite its small size [49,51].

Until the present, more than 2000 discrete plant species have been recorded in Cyprus while the endemism rate of indigenous species reaches approximately 9% [60]. A large proportion of Cypriot plants belongs to the Leguminosae family, but remains uncharted. In an attempt to study the vast genetic resources of agronomically important taxa belonging to the Fabeae tribe, we organized a collection for vetch, lentil, and pea crops wild relatives/landraces. In total, we analyzed 465 plants (155 populations/29 species) by using PI flow cytometry. An estimation of the Cypriot Fabeae genome size variety and the suitability of the technique for Fabeae species characterization was established.

In general, two fluorochromes have been widely employed for determining C-values in plants: DAPI and PI. DAPI is a base-specific dye (AT specific), while PI is an intercalating fluorochrome and has been used to quantify DNA content without the bias of AT/GC base content [61]. Hence, PI has been suggested as the optimal fluorochrome for genome size surveys [62]. In the current study, the adaptation of the LB1 buffer/PI staining protocol proved adequate for the analysis of the Cypriot Fabeae species, providing precise and accurate histograms (CVs lower than 5%) similar to previous studies [25,63,64].

Across the 29 species studied, a remarkable fluctuation of genome size was noted for *Vicia* spp. while *Lens* spp. and *Pisum* spp. were found rather homogeneous (Figure 4*). Vicia cretica* had the smallest genome (1.584 ± 0.007 pg/1C), while *V. faba* DNA content was found to be approximately ten-fold larger, reaching to a mean of 13.829 ± 0.11 pg/1C (Table 1). This great variation in the Fabeae tribe is not unprecedented. Castiglione and colleagues [32] reported that in terms of both chromosome complement and DNA size, C-values in the *Vicia* spp. complex were clearly heterogeneous. Specifically, for species of the Atossa and Wiggersia sections, DNA content had a 2-fold difference despite identical chromosome numbers. In the current study, the majority of taxa could be identified via flow cytometry by using two post-hoc statistic tests (Table 1). Nonetheless, there were cases where DNA size overlapping occurred across lineages.

Still, the relationship among *Vicia* species and its classification has been controversial, since more than 20 taxonomic revisions have been reported since Linneus [65]. For instance, *V. sativa* has been regarded as an aggregate of diverse taxa [26] that was given species rank, while six other taxa were denoted at the subspecies level within *V. sativa* (1. subsp. *amphicarpa* (L.) Batt., 2. subsp. *cordata* (Wulfen ex Hoppe), Asch. & Graebner, 3. subsp. *macrocarpa* (Moris) Arcang., 4. subsp. *angustifolia* L., 5. subsp. *sativa*., 6. subsp. *segetalis* (Thuill.) Gaudin). Indeed, in the current study, *V. sativa* was found to be quite heterogeneous since 1C-values varied from 1.726 to 1.999 pg/1C. Nonetheless, *V. amphicarpa* and *V. angustifolia* were clustered within different groups from *V. sativa* subsp. *sativa* (Table 1), supporting a discrete species assignment. Another taxonomic revision [27] considers *V. narbonensis* as a complex containing *V. johannis*, *V. narbonensis*, and *V. bithynica*. In the current work it was established that, in terms of DNA content, *V. johannis* (6.935 ± 0.014 pg/1C) and *V. narbonensis* (6.949 ± 0.034 pg/1C) were grouped together while *V. bithynica* (3.834 ± 0.035 pg/1C) was clearly demarcated from the former.

Screening of the Kew Plant C-values database revealed that several data presented here are novel (data for *L. ervoides*, *L. nigricans*, *L. orientalis*, *V. cassia*, *V. cretica*, *V. cypria*, *V. laxiflora*, and *V. parviflora*), while other are reported for the first time using flow cytometry (data for *V. amphicarpa*, *V. angustifolia*, *V. bithynica*, *V. ervilia*, *V. hybrida*, *V. johannis*, *V. lathyroides*, *V. lunata*, *V. lutea*, *V. narbonensis*, *V. palaestina*, *V. peregrina*, and *V. pubescens*). A comparison between C-values for species available in the Kew database, as well as the present study was attempted (Figure 5). A high correlation (R² = 0.947) was confirmed across species, except *V. dalmatica*.

Several reasons can cause C-values discrepancies in genome size assessments. Notable variances in flow cytometry protocols, the use of frozen or lyophilized tissues, different fluorochromes, as well as different internal standards [25]. Nonetheless, we found that all six Cypriot *V. dalmatica* accessions had approximately 50% more DNA content (6.872 ± 0.081 pg/1C) compared to previous studies reporting an 1C-value ranging from 3.24 to 4.10 pg [66,67,68]. As a result, such discrepancies cannot be attributed to methods variations but must be based on a diverse/discrete genetic background. *Vicia dalmatica*, is a perennial *Vicia* species in Cyprus and can only be found in the Troodos Mountain region (from 800 m up to 1400 m). As a result, it must sustain extreme conditions, which include cold winter and hot and dry summer with annual temperatures commonly varying from 0 °C to 30 °C. Abiotic stressors have been recently implicated in genome size fluctuation and evolutionary trade-offs [69,70,71,72]. Recently it was also reported that genome size in plants can influence the stress tolerance of invasive and native plants via genomic plasticity [69]. Carta and Angelino also found that plant traits are interconnected with climate seasons and habitat in lilies and that genome size increase is controlled by climate seasonality [73]. Moreover, it was recently established that the Ogre retrotransposon Ty3/gypsy family has a significant part in the genomic size evolution within the Fabeae tribe, causing genomic upsizing [3].

Following C-value estimations, we also attempted to model the DNA size evolutionary trait in correlation to phylogenetic routes, based on internal transcribed spacers. Simulated ancestral states grounded on parsimony were depicted showing a complex evolutionary history (Figure 6). Based on C-value character development it seems that genomic size fluctuated independently to the evolution and diversification of the Fabeae species. The main genomic augmentation was found in the clade of *V. faba* and *V. peregrina* that were also genetically affiliated. In contrast, there were also instances when a genomic downsizing from ancestral nodal routes was evident. Independence of C-values and genetic relationships has also been reported for *Vicia* spp. at the karyotypic level.

The basic numbers of *Vicia* chromosomes are X = 5, 6, or 7. Thus, there is an exclusive variety and karyotypic diversity across diploid taxa (2n = 2x = 10, 2n = 2x = 12, and 2n = 2x = 14, respectively), making the genus a unique model of karyotypic evolution [74]. However, plants with different basic chromosome numbers exist even at the species level (all basic numbers of chromosomes have been reported for *V. sativa*, *V. amphicarpa*, and *V. lathyroides*) [75].

Concluding, the DNA content reported for several taxa of the Fabeae tribe of Cyprus can be used as a reliable standard and contribution to a further depiction of the evolutionary associations amongst these agronomically important species. Moreover, these C-values can be a guide map of breeding efforts regarding such distinctive germplasm. Despite the fact that Leguminosae taxa belong to one of the most widespread families, there are still several gaps regarding our knowledge on legumes and crop wild relatives (CWRs). Specifically, focus on local CWRs and their extraordinary genetic diversity/genome size variation could facilitate precise and imminent decisions on plant genetic resources utilization and preservation.

## Figures and Tables

**Table 1:** List of Fabeae taxa (in alphabetic order), number of analyzed accessions, DNA content reported in Kew database, internal standards used, predicted Mbps, mean 1C-values with standard deviations, coefficient of variance, ranges of DNA content, HSD and LSD tests. Nomenclature is according to Hand et al., 2011 [51].

Species	No. of Accessions	DNA Content/Data from Kew (pg/1C)	Internal Standard ^3^	Mbp ^4^	Mean DNA Content ± SD (pg/1C)	%CV	Range of DNA Content (pg)	HSD Test ^5^	LSD Test ^5^
*L. culinaris*	18	4.21	*Z. m*	4260.983	4.357 ± 0.05	1.148	4.209–4.448	ij	k
*L. ervoides* ^1^	5	-	*Z. m*	3391.313	3.468 ± 0.026	0.750	3.437–3.494	l	n
*L. nigricans* ^1^	3	-	*Z. m*	3452.992	3.531 ± 0.015	0.425	3.521–3.548	l	n
*L. orientalis* ^1^	2	-	*Z. m*	4280.706	4.377 ± 0.062	1.416	4.333–4.421	hij	jk
*P. fulvum*	2	4.7	*Z. m*	4735.476	4.842 ± 0.035	0.723	4.817–4.867	g	g
*P. sativum* subsp. *biflorum*	3	4.75	*Z. m*	4409.802	4.509 ± 0.091	2.018	4.437–4.611	hi	hi
*P. sativum* subsp. *sativum*	8	4.9	*Z. m*	4422.272	4.522 ± 0.026	0.575	4.493–4.572	h	h
*V. amphicarpa* L. ^2^	2	1.65	*S. l*	1617.612	1.654 ± 0.059	3.567	1.612–1.696	qr	t
*V. angustifolia* L. ^2^	7	2.01	*G. m*	1964.662	2.009 ± 0.061	3.036	1.929–2.094	p	r
*V. bithynica* L. ^2^	3	3.83	*Z. m*	3749.978	3.834 ± 0.035	0.913	3.808–3.874	k	m
*V. cassia Boiss* ^1^	6	-	*Z. m*	4343.298	4.441 ± 0.128	2.882	4.356–4.694	hi	ij
*V. cretica Boiss. & Heldr.* ^1^	1	-	*S. l*	1549.096	1.584 ± 0.007	0.442	1.577–1.591	r	t
*V. cypria Kotschy* ^1^	1	-	*P. s*	5368.995	5.49 ± 0.022	0.401	5.467–5.51	f	f
*V. dalmatica A. Kern.*	6	6.87	*P. s*	6720.816	6.872 ± 0.081	1.179	6.716–6.94	d	d
*V. ervilia* L. ^2^	8	4.2	*Z. m*	4154.789	4.248 ± 0.025	0.589	4.22–4.301	j	l
*V. faba* L.	14	13.62	*S. c*	13,525.042	13.829 ± 0.11	0.795	13.66–13.98	a	a
*V. hybrida* L. ^2^	11	6.78	*P. s*	6546.110	6.693 ± 0.118	1.763	6.542–6.971	e	e
*V. johannis Tamamsh* ^2^	1	6.94	*P. s*	6782.479	6.935 ± 0.014	0.202	6.919–6.943	d	d
*V. lathyroides* L. ^2^	3	2.53	*G. m*	2477.600	2.533 ± 0.05	1.974	2.484–2.583	n	p
*V. laxiflora Brot.* ^1^	2	-	*G. m*	2762.850	2.825 ± 0.003	0.106	2.823–2.827	m	o
*V. lunata (Boiss. & Balansa) Boiss.* ^2^	5	1.83	*S. l*	1750.033	1.789 ± 0.022	1.230	1.768–1.824	q	s
*V. lutea* L. ^2^	1	7.4	*P. s*	7143.214	7.304 ± 0.011	0.151	7.291–7.312	c	c
*V. narbonensis* L. ^2^	4	6.95	*P. s*	6795.878	6.949 ± 0.034	0.489	6.912–6.994	d	d
*V. palaestina Boiss.* ^2^	1	2.24	*G. m*	2191.757	2.241 ± 0.019	0.848	2.223–2.26	o	q
*V. parviflora Cav.* ^1^	3	-	*G. m*	2560.078	2.618 ± 0.08	3.056	2.526–2.673	mn	p
*V. peregrina* L. ^2^	6	8.97	*P. s*	8773.149	8.971 ± 0.069	0.769	8.862–9.038	b	b
*V. pubescens (DC.) Link* ^2^	6	2.79	*G. m*	2730.739	2.792 ± 0.062	2.221	2.667–2.831	m	o
*V. sativa* L. subsp. *sativa*	17	2.25	*S. l*	1756.891	1.796 ± 0.063	3.508	1.726–1.999	q	s
*V. villosa* subsp. *eriocarpa (Hausskn.) P. W. Ball*	6	2.28	*S. l*	1782.568	1.823 ± 0.075	4.114	1.748–1.947	q	s

^1^ First report in the Fabeae literature (according to Kew Plant C-values database https://cvalues.science.kew.org/ (accessed on 3 March 2023)); ^2^ First report using flow cytometry; ^3^ *Z. m*: *Zea mays* cv. CE-777 (2.715 pg/1C); *S. l*: *Solanum lycopersicon* cv. Stupické polní tyčkové rané (0.98 pg/1C); *G. m*: *Glycine max* cv. Polanka (1.25 pg/1C); *S. C*: *Secale cereale* cv. Dankovské (8.095 pg/1C); *P. s*: *Pisum sativum* cv. Ctirad (4.545 pg/1C); ^4^ (1 pg = 978 Mbp) as reported in Dolezel [54]; ^5^ Species having identical lowercase letters were not found significantly different at *p* = 0.05.

## Data Availability

All relevant data are within the paper and its Supplementary Materials.

## Supplementary Materials

The following supporting information can be downloaded at: https://www.mdpi.com/article/10.3390/plants12071469/s1, Table S1: Accessions, C-values and Coordinates of the Fabeae taxa; Supplementary Figure S1: Residuals analysis for the Fabeae species.
