# Supplementary material for: Genome Size Variation across a Cypriot Fabeae Tribe Germplasm Collection
Source: Plants (Basel). 2023 Mar 27;12(7):1469. doi: 10.3390/plants12071469 (PMC10096862; doi:10.3390/plants12071469)
Supplement: Supplementary file 1 [file plants-12-01469-s001.zip › plants-2291282 supplementary.pdf]

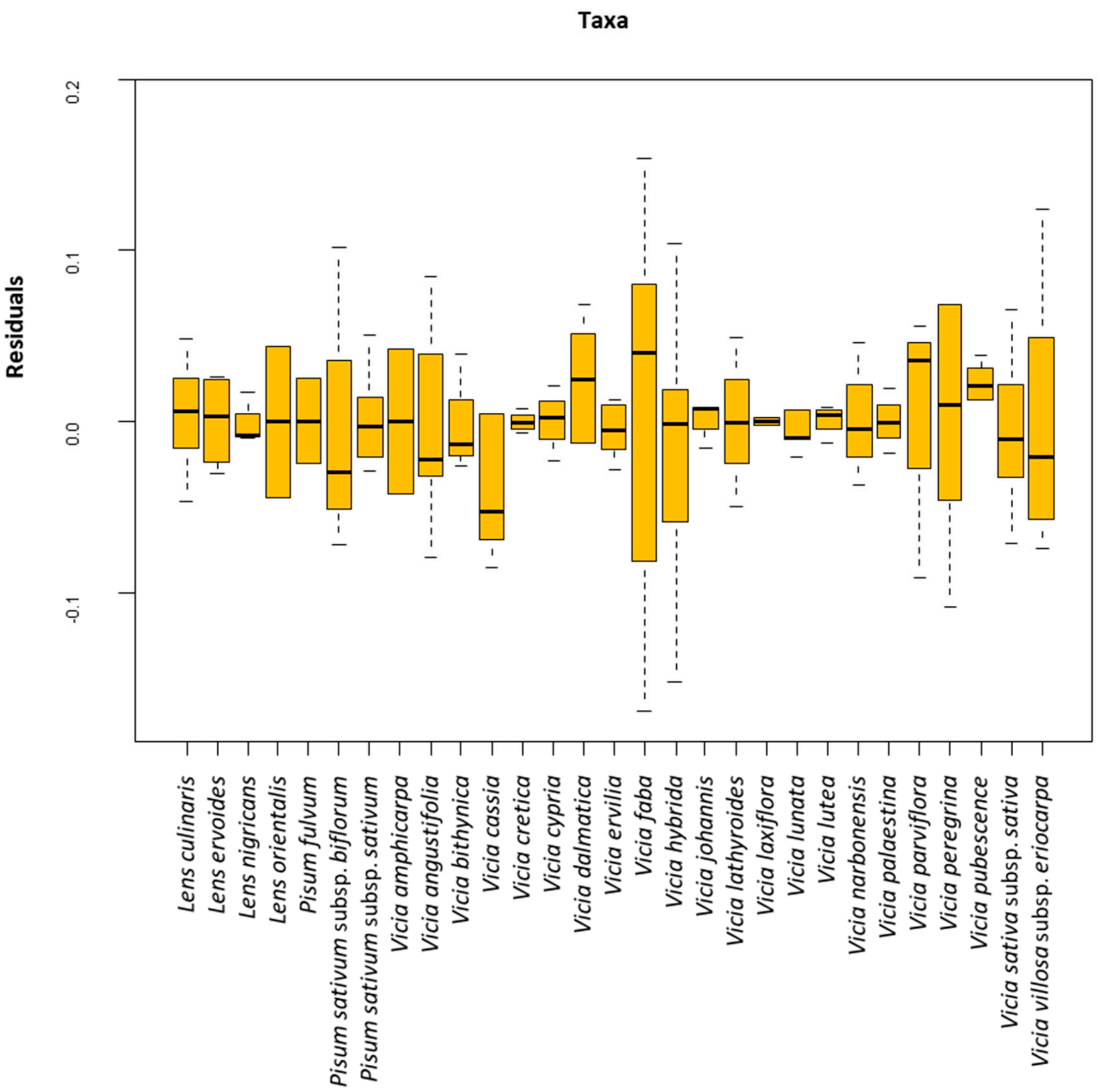

Supplementary Figure S1. Residuals analysis across species. A regression function, as well as independent and identically distributed errors were found to be consistent (means are positioned at the zero axis).
